# How to engage patients in achieving patient safety: A qualitative study from healthcare professionals’ perspective

**DOI:** 10.1016/j.heliyon.2023.e13447

**Published:** 2023-02-07

**Authors:** Ekorini Listiowati, Amal Chalik Sjaaf, Anhari Achadi, Adang Bachtiar, Merita Arini, Elsye Maria Rosa, Yuyun Pramayanti

**Affiliations:** a Department of Family Medicine and Public Health, School of Medicine, Faculty of Medicine and Health Sciences, Universitas Muhammadiyah Yogyakarta, Yogyakarta, Indonesia; b Master of Hospital Management, Postgraduate Program, Universitas Muhammadiyah Yogyakarta, Yogyakarta, Indonesia; c Department of Health Policy and Administration, Faculty of Public Health, Universitas Indonesia, Indonesia

**Keywords:** Developing countries, Patient engagement, Patient safety, Safety culture, Quality of care, PE, patient engagement, PS, patient safety, HCP, healthcare professional, HCR, healthcare recipient, PAR, participatory action research, SOP, standard operating procedure

## Abstract

All parties involved in health care, including patients and their families/caregivers play a significant role to achieve patient safety. Furthermore, patient engagement (PE) has not been adequately implemented to achieve safe healthcare in Indonesia, despite the introduction of the patient-centered care paradigm. This study aims to explore healthcare professionals' (HCPs) perspectives on PE and its application technique. A qualitative study was conducted in the chronic wards of a faith-based private hospital in Yogyakarta Province, Indonesia. Four focus group discussions among 46 HCPs, followed by 16 in-depth interviews, were carried out. Furthermore, the verbatim transcripts were subjected to thematic analysis. The result showed four main themes, including PE as a strategy for achieving safe healthcare, factors affecting its implementation, the need for comprehensive strategies to engage the patients, and their roles in safety efforts. Furthermore, the implementation of PE can be enhanced by encouraging healthcare professionals (HCPs) to play proactive roles in empowering recipients. To achieve PE, “partnership culture” and the removal of potential barriers as well as determining factors, must be established. This requires a high-level commitment, organizational support with a top-down approach, and integration into healthcare systems. In conclusion, PE is essential for patient safety and can be enhanced by strengthening organization support, integrating into the healthcare system, improving HCPs’ roles, and empowering patients and caregivers to overcome potential barriers.

## 1 Introduction

Patient safety (PS) is a global concern to ensure the provision of high-quality healthcare. Over 80% of safety incidents experienced by patients are preventable by fostering continuous safety culture improvement [1] In the health systems of developing countries, a patient safety culture characterized by transparency, communication, teamwork, and strong leadership is essential to ensure that patients receive reliable and safe care [2]. Moreover, other unfavorable factors, such as staffing shortages, insufficient structures and overcrowding, a lack of healthcare supplies, and inadequate hygiene and sanitation, contribute to unsafe healthcare delivery in developing countries [3]. A thorough and comprehensive approach is necessary to effectively manage this situation [4].

Since Patient engagement (PE) has become essential to be enhanced, WHO placed the “patient for patient safety program” at the core of the latest strategies to achieve PS and higher quality health services, PE has become essential to be enhanced [5,6]. Several studies found that employing patient and family engagement strategies reduced hospital-acquired infections, medical errors, serious safety accidents, and increased patient satisfaction [[7], [8], [9]]. Although these initiatives are highly beneficial, they are often confined to policy development, administrative procedures, patient education, increasing healthcare professionals' (HCPs') awareness and capacity, as well as technical assistance. Furthermore, specific initiatives to engage patients in risk reduction remain a challenge, as indicated by the preponderance of studies indicating low levels of engagement [4,7,10,11]. Hospitals typically continue to highlight their opportunities and HCPs’ preferences when developing strategies for patient safety improvement without considering their perspective and needs [12].

Recently, the implementation of PE in Indonesia has been insufficient to ensure safe healthcare. For instance, the hospital accreditation guidelines issued by the Ministry of Health, Republic of Indonesia, do not include PE as a strategy for improving safety. However, as indicated by the World Health Organization, the patient should be placed in the center of care as the national hospital accreditation program was introduced [6]. Based on the background, this study aims to explore the perception of hospital HCPs about PE, including their concerns, experiences with existing practices, barriers, and potential strategies for its implementation to achieve patient safety. This study is expected to provide useful results for various stakeholders, including hospital managers, health workers, and the government, by providing insights related to increasing PE as a strategy to improve their safety.

## 2 Methods

### 2.1 Study setting

This qualitative study was conducted in a faith-affiliated private hospital in Sleman District, Yogyakarta Special Region Province, Indonesia, from November 2020- to April 2021. This nationally accredited type C hospital has 216 beds with 15 specialists and subspecialist services. The study was restricted to the context of chronic care inpatient services due to their long-term needs and access to health care. Furthermore, chronic patients are more susceptible to experiencing substandard healthcare and patient safety problems [13,14].

### 2.2 Study design

A qualitative study was conducted with the PAR (participatory action research) technique to explore ways to engage patients to achieve safety. This technique focuses on studies intended to facilitate actions, drawing on the paradigms of critical theory and constructivism [15]. The first part of PAR was reported in this article, namely diagnosing stage that used a descriptive-explorative approach to investigate PE implementation based on HCPs’ perspectives.

### 2.3 Participants

The informants were HCPs involved in chronic care and were purposively selected using criterion sampling from the list of hospital staff. The nursing staff were approached by research assistant #1. All of participants were agreed to participate in this study. Furthermore, the HCPs were divided into four Focus Group Discussion (FGD) groups for nurses with less than 2 years (n = 9) work periods, more than 2 years (n = 10), supervisor nurses (n = 15), and managers (nurses and general practitioners) (n = 8) (Table 1). Twelve nurses from four FGDs with rich information and four specialist physicians were subjected to in-depth interviews (IDIs). Due to the greater involvement of nurses in the treatment of long-term patients on the ward, the number of nurse informants was more substantial than that of other HCPs.

**Table 1:** Demographics of participants.

Group	Informant's Code	Gender	Age (year)	Work period (year, month)	Education Level
Functional nurses, work period <2 years (n = 9)	#1	F	27	0.9	Undergraduate
	#2	F	27	0.9	Undergraduate
	#3	M	29	0.9	Undergraduate
	#4	F	28	1.7	Undergraduate
	#5	M	28	1.7	Undergraduate
	#6	F	26	0.9	Undergraduate
	#7	F	27	1.7	Diploma 3
	#8	F	25	1.7	Diploma 3
	#9	F	27	1.7	Undergraduate
Functional nurses. Work period >2 years (n = 10)	#10	M	30	5.6	Undergraduate
	#11	F	32	5.11	Undergraduate
	#12	F	29	2.7	Undergraduate
	#13	F	35	11.5	Diploma 3
	#14	M	32	5.6	Undergraduate
	#15	F	33	9.4	Diploma 3
	#16	F	29	3.4	Undergraduate
	#17	F	30	5.11	Undergraduate
	#18	F	36	10.11	Diploma 3
	#19	M	29	5.10	Diploma 3
Supervisor nurses (n = 15)	#20	F	41	19.0	Diploma 3
	#21	F	34	7.5	Undergraduate
	#22	M	52	30.11	Diploma 3
	#23	F	47	22.2	Diploma 3
	#24	F	33	7.5	Undergraduate
	#25	F	54	29.4	Undergraduate
	#26	M	46	19.4	Undergraduate
	#27	F	32	5.11	Undergraduate
	#28	F	32	7.1	Undergraduate
	#29	M	43	19.7	Undergraduate
	#30	F	31	5.11	Undergraduate
	#31	M	49	23.4	Undergraduate
	#32	F	32	6.9	Undergraduate
	#33	F	45	19.1	Diploma 3
	#34	M	34	19.1	Undergraduate
Specialist Physicians (n = 4)	#35	M	53	13.4	Postgraduate, internist
	#36	M	47	15	Postgraduate, neurologist
	#37	F	54	11	Postgraduate, internist
	#38	M	52	12	Postgraduate, internist
Managers (n = 8)	#39	M	50	30	Undergraduate, nurse
	#40	F	40	15	Postgraduate, nurse
	#41	F	46	21	Postgraduate, pharmacist
	#42	F	33	7	Undergraduate, general practitioner
	#43	M	44	19.11	Undergraduate, nurse
	#44	F	46	23.4	Postgraduate, midwife
	#45	M	34	07.7	Undergraduate, general practitioner
	#46	F	47	26.2	Undergraduate, nurse

### 2.4 Data collection

An online focus group and in-depth interviews were conducted using an internet conference meeting platform. Online focus groups are a development that resulted from the Internet's adaption of existing methodologies [16]. FGD and interview guides were used to facilitate the exploration of information and took about an hour and a half. Furthermore, questions were focused on “the perspectives of HCPs on the importance of PE, existing practices of PE, and concerns, potential barriers, and strategies to improve its implementation.**”** In this study, in-depth interviews were performed after all FGD sessions. In-depth interviews aim to explore further findings in the discussion that needs to be explored in more depth, as well as sensitive topics that participants may not want to address in a group environment [17]. IDIs were conducted with 14 patients and 15 caregivers, as the data triangulation. Triangulation was conducted for data credibility among different groups of informants and member-checking. An audit trail was then provided to verify trustworthiness by maintaining a thorough record of the data-collecting process using field notes, a daily logbook/journal, and the safe storage of interview recorders.

Interviews and FGDs were conducted by EMR as a moderator in the Indonesian language and partly in a mixture of regional languages (Javanese) and recorded. The principal investigator/PI (EL) presented as an observer, and YP as the note-taker. Afterward, IDIs were conducted with specialist physicians by EL and YP.

### 2.5 Data analysis

Each IDIs and FGDs were transcribed verbatim by study assistant #2 immediately after each data collection process. All transcripts were managed and coded using NVivo 12+, contributing to the study's dependability and confirmability. Furthermore, MA, YP, and study assistant #2 maintained data credibility by rechecking before and during the coding. Thematically, data coding was performed by MA to get selective and axial coding, as well as themes [18]. The analysis for focus group discussion was conducted in the following steps: first, the discussion transcripts were thoroughly scrutinized. The reading and re-reading process enabled the study to familiarize itself with the data and understand the larger picture. Second, initial codes were generated inductively for each discussion and compared with those of other discussions to identify recurring codes. Third, essential groups/categories of the identified codes were created, and each group was assigned a specific theme. The codes and themes were reviewed and discussed among EL and MA throughout the analysis. Furthermore, the data analysis was performed continuously after each FGD session. The same three processes with the FGD have been implemented for the analysis of in-depth interviews. A saturation evaluation was conducted in which data collection was stopped after data saturation was reached during the analysis, and no new categories were found. Saturation is the point at which it is unlikely to obtain any additional concept-relevant insights [19].

EL, MA, YP, and EMR, who conducted data collection and analysis, have a background in hospital management studies. All of them are female and have a formal educational basis of health and qualitative study methodology. Two study assistants with a health education background were trained according to their respective roles, including informants, recruiters, and transcribers. Furthermore, ACS, AA, and AB were the supervisors and provided valuable theme formulation and manuscript writing input. This study is reported as per consolidated criteria for reporting qualitative checklist (COREQ) to ensure quality [20].

### 2.6 Ethics

Ethical approval was obtained from the Research and Community Engagement Ethical Committee, Faculty of Public Health, Universitas Indonesia (No: Ket-584/UN2·F10. D11/PPM.February 00, 2020) before the commencement of the study. Furthermore, the researcher EL communicated with the hospital manager about the research project. The informants were given explanation of the study and conflict of interest in research prior to study. Written consent was obtained before data collection. Furthermore, the informant's confidentiality was assured by replacing all personal identities with unique transcripts and report document codes.

## 3 Results

**Fig. 1:**
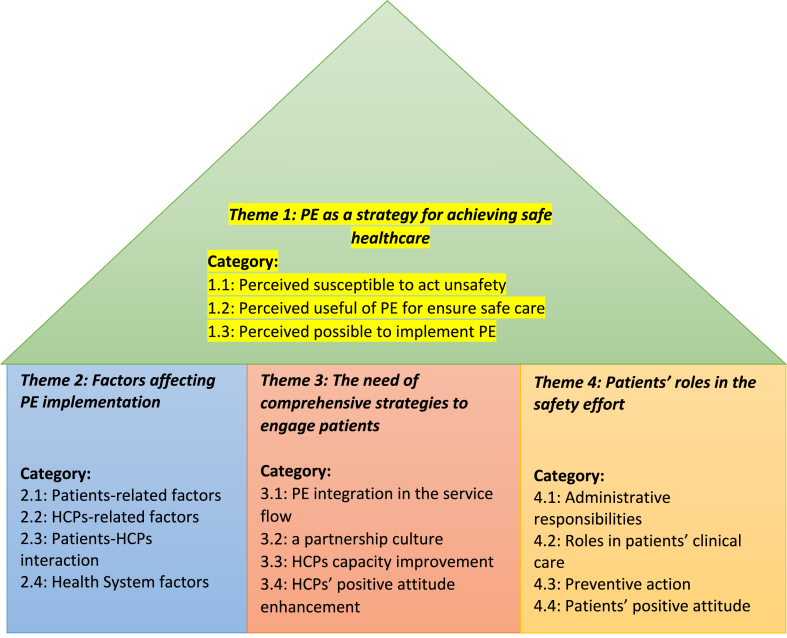
Themes emerging from data analysis.

As indicated in Table 1, all informants were permanent hospital employees. The majority of HCPs were female (65.2%, n = 30), with a minimum, average, and maximum age of 25, 37.17, and 54 years old, respectively. The mean work period of 10 years 11 months (min. 9 months, max. 30 years 11 months). Four main themes emerged from this study, as presented detailed in Fig. 1.

### 3.1 Theme 1: PE as a strategy for achieving safe healthcare

All informants agreed that PE is one of the important factors in enhancing the overall quality of treatment, especially patient safety. They also pointed out that everyone in the hospital is susceptible and at risk of a safety incident. Despite obstacles that should be overcome, they emphasized that PE might become one of the methods for enhancing patient safety. Informant HCPs also believed that implementing PE is possible.

The active participation of the patient or the family helps us in providing nursing care, for instance, in the case of medication. We are three to four nurses on duty, although the patients we treat are dozens. Furthermore, we have experience with the wrong distribution of oral drugs due to the similarity of patients' names. Then they confirmed the medicine we gave had been switched. Thus, the involvement of the patient is very necessary. (Informant #3)

Patients participation is very important because with their knowledge and understanding, there will be good coordination of all health services, starting from admission, diagnosis, planning, and treatment, and even after they are dischared. (Informant #13)

### 3.2 Theme 2: factors affecting PE implementation

Complex factors from patients, HCPs, patients-HCP interactions, and the healthcare system can potentially affect PE application. They facilitate and impede the operation of this concept in the hospital. Based on patients’ perspectives, barriers associated with their education and health literacy, culture, willingness to be involved, attitude, health conditions, and characteristics influence their readiness to participate in safe healthcare. Meanwhile, staff perspectives, preferences, capacity, and workload influence their existing practices in engaging patients. Other factors that affect partnership patterns during care services include gaps of interests, needs, and socio-demographic differences between HCPs and patients.

Based on the patient’s perspective, cultural factors play a significant role. Generally, the Javanese patient is submissive. Then they think, “Ah, officers are already experts, how could I teach them?” Therefore, I think it depends more on that factor. Suppose I had administered the medicine, almost every patient would have believed that his medicine was true. (Informant #3)

PE is good, but in my opinion, the ones who are not ready are our patients. Their knowledge and comprehension of this disease are too unsophisticated. Moreover, sometimes his family had another concern, and they did not understand the problem. (Informant #36)

The healthcare system's policy concerning PS, workflows, coordination between units, human resources management, and building design had several impacts on PE implementation. The informants expressed these situations in the following quotations.

Due to demanding work situations, nurses are required to perform additional tasks, resulting in inappropriate conversational conduct or explanations to patients. (Informant #26)

As healthcare providers, hospitals have restricted access to information about errors, such as medication-related, wrong patients, and procedures. Unless it has already become a media sensation, the hospital will undoubtedly cover it up. Consequently, the patient’s understanding of the situation fails to focus on the essence of the risk but rather on the uproar. (Informant #41)

Informants had mixed feelings regarding their belief in the importance of PE against the impacts of patients’ speaking up and other proactive roles.

Occasionally, what happens is annoying. Now is the era of being completely online. They are already looking for their references by themselves. Thus, there are positive and negative impacts of PE implementation. (Informant #35)

I apologize because I do not like being reminded by patients. Suppose the patient reminds us of patient safety procedures, we will not implement them. Therefore, there is a mistake when the patient reminds us. (Informant #43)

The question is whether the hospital is ready to be more open to patients regarding safety concerns. (Informant #41)

### 3.3 Theme 3: the need for comprehensive strategies to engage patients

A multifaceted approach is required in enhancing PE. The informants emphasized the necessity to incorporate PE initiatives into the care service flow, especially by including PE activities in pre-existing standard operating procedures (SOPs), initial patient orientation, and strengthening interprofessional collaborative care implementation. HCPs also highlighted the pivotal role of therapeutic communication and health promotion with a high-quality approach to build partnerships with patients and family/caregivers and support their empowerment efforts. Furthermore, they observed that patients with diverse socio-demographics require problem-solving, motivation, and a sense of safety to participate in care services.

Because involving patients in safety is a technical matter, I think the regulations should be incorporated into standard operating procedures. In the SOPs of new inpatients, nurses can provide added explanations about the importance of their involvement in realizing the six safety goals. For example, “Mom … If the nurse injects a medicine without obtaining your name and date of birth, please remind him/her.” (Informant #39)

The provision (educational material) to patients in leaflets or posters and reading materials about patient safety in the lay language is important. So he knows, “Oh, situations like this have the potential to harm me.” It includes a form of effective communication that many people may understand, for example, with symbols. (Informant #36)

Effective HCPs' capacity improvement using innovative methods was a crucial prerequisite of PE. HCPs are expected to adopt a new paradigm regarding the importance of patients’ involvement in healthcare by increasing awareness sessions and providing training for improving knowledge and skill in communication and education.

The evaluation result of this study indicates that our information dissemination is less innovative. Maybe we can use media such as radio, social media, or in the form of jokes. Therefore, it can be formed in a more engaging manner. SOP is important because it is an approach for implementation, but socialization that is easily understood and accepted is considerably more crucial. (Informant #41)

These comprehensive approaches were vital for establishing a culture of patient engagement. It requires strong leadership and an integrated health service delivery system to ensure the implementation of a safety culture. Moreover, the implementation of religious values was cited as a vital effort to create a comfortable atmosphere.

Based on the care provider’s perspective, what should be built is a safety culture and how to construct concepts, culture, and beliefs about the importance of patient safety. It includes developing the behavior that will become one of our commitments to provide safe patient care if we are reminded. (Informant #39)

The key is to build leadership on a top-down basis. Then, good leadership should be improved, followed by systems and procedures. Furthermore, the reward and punishment mechanism must be applied. (Informant #41)

Normatively, as a faith-based hospital deciding to implement the shari’ah principle, these values could be included in campaign activities, socialization, and fostering the spirit that “when I act for patient safety, it is to prevent harm”. This act has a reward, and it is our faith teaching. (Informant #41)

### 3.4 Theme 4: patients’ roles in the safety effort

Informants identified the crucial roles of patients to be involved in safety efforts. Patients should fulfill their administrative responsibilities and participate as much as possible during almost all clinical service stages. Furthermore, positive patient attitudes such as cooperation, active questioning, initiative, honesty, and compliance with HCP advice may facilitate the application of PE. Informants acknowledge that patients can take precautionary measures, such as confirming their identity, notifying HCPs of planned surgery, and taking other precautions to prevent injury.

Patient involvement in patient safety efforts provides accurate, complete, clear, and honest information, and they are aware of their responsibilities. Additionally, he may ask questions he does not comprehend in order to receive health care. They should also understand and comply with hospital regulations, as well as financial obligations that must be fulfilled. (Informant #13)

## 4 Discussion

This study creates a more effective attempt to be the first to improve the implementation of hospital PE in Indonesia by thoroughly exploring HCPs' perspectives and understanding their experiences, expectations, and concerns. It described HCPs’ initial acceptance of the PE concept and revealed their perspectives of its importance as a strategy for building safe healthcare. Conversely, multilayered constraints in performing PE were identified. This study identified crucial and comprehensive approaches for enhancing PE within the health system and the individual domain.

The result shows that all informants agreed that PE is one of the essential components in enhancing the overall quality of health care, especially patient safety. Furthermore, a previous study showed that patient-family engagement offers a promising pathway toward better quality, more efficient, and improved population health [21]. Three areas have been the focus of the attempt to involve the patient in safety efforts. They include enlisting patients in detecting adverse events, empowering them to ensure safe care, and emphasizing their involvement as a means of improving safety culture [22].

Furthermore, this study reflected existing obstacles in the patients' and caregivers’ aspect, HCPs, and the healthcare system that must be addressed. HCPs emphasize the unreadiness to engage due to low health literacy and cultural barriers from the perspective of patients. These results are consistent with the study on the health literacy of Indonesian and how certain tribal cultural backgrounds might influence the willingness of patients to speak up [[23], [24], [25], [26]]. Meanwhile, the challenges facing HCPs and healthcare system aspects were dominated by workload problems and the absence of health facility policies to engage patients. In addition to staff awareness and capacity, these barriers are similar to those identified in previous studies in several countries [[27], [28], [29]].

This study's results have important practical implications. In order to overcome these complex barriers, it has been discovered that a culture of patient safety must be developed, which is the more it is implemented, the more accelerated PE could be applied, and vice versa. It is suggested that a top-down regulation, supported by planning management, is essential to guarantee an integrated healthcare system that places greater emphasis on a conducive atmosphere for HCPs to work and patients to speak up. These strategic approaches are required for the cultural transformation from conventional healthcare services to a more partnership culture. Furthermore, human resource shortages must be managed through the establishment of appropriate policies, redesigning service flows and structures, the provision of technical assistance, and the implementation of various innovative intervention methods to facilitate PE. Therefore, there is a need to improve awareness and HCPs' capacity. These strategies are essential, considering their vital roles in health care delivery as educators and advocators for the patients [8]. As a prerequisite for implementing PE, they are also expected to increase their commitment to being more proactive in promoting PE, adopt a more positive attitude, and be more responsive to patient input.

The significant implication of these results is that the empowerment of patients and families/caregivers in patient safety and involvement in health services cannot be achieved alone during hospital service delivery. A previous study shows that it requires the role of many parties from the macro system to the individual level [7,8,26]. In line with this study result, the WHO highlighted attempts to empower and facilitate patients and their families to be advocated for change collaboratively [6]. Consequently, it is recommended that continual efforts be made to strengthen collaboration between patients, HCPs, policymakers, payers (health insurance), community, and health education institutions to resolve these cascading barrier [8,23]. Safer healthcare requires a high-level commitment, in addition to strengthening global leadership and expanding to the institutional level, a non-blaming culture with a commitment to learning from errors and patient experiences, transparency in communication, and commitment to strengthening HCPs and community education [5,6].

As an approach to strengthening safety, this study's informants recommend that many roles should be taken by the patient, including improving safety culture and empowering patients and their families to ensure safe care. A previous study showed that efforts to engage patients in safety efforts focused on three areas. They include enlisting patients in detecting adverse events, empowering them to ensure safe care, and emphasizing their involvement as a means of improving the culture of safety [22]. This study result showed that patients can take a role in the administrative process, such as confirming their identity and alert HCPs to an upcoming procedure. Furthermore, PE can improve documentation and scheduling accuracy [30].

## 5 Limitations and strengths

This study has limitations regarding the contextualized setting and other factors. Notably, it was conducted in the context of Islamic-based hospitals in Yogyakarta, Indonesia, where the majority of the population is Javanese and Muslim. Other institutions in different regions with diverse socio-demographics might face unique circumstances and challenges. Furthermore, different backgrounds might produce diverse emphasized study results. Despite these limitations, the study provides valuable insights into initial PE development in low resources countries. Furthermore, HCPs were included with a wide variety of expertise, which broadened the range of perspectives as a strength of this study.

## 6 Conclusion

Conclusively, PE is crucial to providing high-quality care and ensuring patient safety based on HCPs' points of view. This study showed complex hurdles, including patients, hospital staff, the relationship between healthcare recipients and HCPs, and the healthcare system. However, the most significant concern identified was patient unreadiness. By empowering patients and family/caregivers, boosting the proactive roles of HCPs, integrating PE into the healthcare system, and increasing organizational support, PE programs could be reinforced. Consequently, a high-level commitment and top-down approach are essential for overcoming potential hurdles and factors influencing them. Further studies should assess PE implementations’ safety, effectiveness, and incremental cost in developing countries.

## Author contribution statement

Ekorini Listiowati, Amal Chalik Sjaaf, Anhari Achadi, Adang Bachtiar: Conceived and designed the research; Performed the research; Analyzed and interpreted the data; Wrote the paper. Merita Arini, Elsye Maria Rosa, Yuyun Pramayanti: Performed the research; Analyzed and interpreted the data; Wrote the paper.

## Funding statement

This study was supported by Universitas Muhammadiyah Yogyakarta.

## Data availability statement

Data will be made available on request.

## Prior presentation and publication

This research has never been published in any form.

## Additional information

No additional information available for this paper.

## Declaration of competing interest

The author(s) declared no potential conflicts of interest in respect to the research, authorship, and/or publication of this article.

## References

[1] World Health Organization (2020). https://www.who.int/publications/i/item/9789240010338.

[2] Elmontsri M., Banarsee R., Majeed A. (2018). Improving patient safety in developing countries – moving towards an integrated approach. JRSM Open.

[3] World Health Organization (2017). https://apps.who.int/iris/bitstream/handle/10665/255507/WHO-HIS-SDS-2017.11-eng.pdf?sequence=1&isAllowed=y.

[4] NHS England and NHS Improvement (2019). https://www.england.nhs.uk/wp-content/uploads/2020/08/190708_Patient_Safety_Strategy_for_website_v4.pdf.

[5] World Health Organization (2016). http://apps.who.int/bookorders.

[6] World Health Organization (2013). https://cdn.who.int/media/docs/default-source/patient-safety/pfps/pfps_brochure_2013.pdf?sfvrsn=45a18595_7.

[7] Kim J.M., Suarez-Cuervo C., Berger Z., Lee J., Gayleard J., Rosenberg C., Nagy N., Weeks K., Dy S. (2017). Evaluation of patient and family engagement strategies to improve medication safety. The Patient - Patient-Centered Outcomes Research.

[8] (2015). A report of the NCIOM task force on patient and family engagement, issue brief: patient and family engagement: a partnership for culture change. N. C. Med. J..

[9] Duhn L., Godfrey C., Medves J. (2020). Scoping review of patients' attitudes about their role and behaviours to ensure safe care at the direct care level. Health Expect..

[10] Hammoud S., Amer F., Lohner S., Kocsis B. (2020). Patient education on infection control: a systematic review. Am. J. Infect. Control.

[11] Trier H., Valderas J.M., Wensing M., Martin M., Egebart J., Martin H.M. (2015). Involving patients in patient safety programmes: a scoping review and consensus procedure by the LINNEAUS collaboration on patient safety in primary care. Eur. J. Gen. Pract..

[12] Newell S., Jordan Z. (2015). The patient experience of patient-centered communication with nurses in the hospital setting: a qualitative systematic review protocol. JBI Database System Rev Implement Rep.

[13] Grover A., Joshi A. (2015). An overview of chronic disease models: a systematic literature review. Global J. Health Sci..

[14] Smith S.M., Wallace E., O'Dowd T., Fortin M. (2016). Interventions for improving outcomes in patients with multimorbidity in primary care and community settings. Cochrane Database Syst. Rev..

[15] Baum F., MacDougall C., Smith D., Baum P.F. (2006). Participatory action research. J. Epidemiol. Community Health.

[16] Nyumba T.O., Wilson K., Derrick C.J., Mukherjee N. (2018). The use of focus group discussion methodology: insights from two decades of application in conservation. Methods Ecol. Evol..

[17] Gill P., Stewart K., Treasure E., Chadwick B. (2008).

[18] Vaismoradi M., Turunen H., Bondas T. (2013). Content analysis and thematic analysis: implications for conducting a qualitative descriptive study. Nurs. Health Sci..

[19] Morse J.M. (1995). The significance of saturation. Qual. Health Res..

[20] Tong A., Sainsbury P., Craig J. (2007). Consolidated criteria for reporting qualitative research (COREQ): a 32-item checklist for interviews and focus groups. Int. J. Qual. Health Care.

[21] Carman K.L., Dardess P., Maurer M., Sofaer S., Adams K., Bechtel C., Sweeney J. (2013). Patient and family engagement: a framework for understanding the elements and developing interventions and policies evidence & potential. February.

[22] Agency for Healthcare Research and Quality (2019).

[23] Chegini Z., Janati A., Babaie J., Pouraghaei M. (2020). Exploring the barriers to patient engagement in the delivery of safe care in Iranian hospitals: a qualitative study. Nurs Open.

[24] Domecq J.P., Prutsky G., Elraiyah T., Wang Z., Nabhan M., Shippee N., Brito J.P., Boehmer K., Hasan R., Firwana B., Erwin P., Eton D., Sloan J., Montori V., Asi N., Moain A., Dabrh A., Murad M.H. (2014). Patient engagement in research: a systematic review. BMC Health Serv. Res..

[25] Mubarokah N.K. (2018). Health literacy and health behavior in the rural areas. KnE Life Sciences.

[26] McCormack L., Thomas V., Lewis M.A., Rudd R. (2017). Improving low health literacy and patient engagement: a social ecological approach. Patient Educ. Counsel..

[27] Davis R.E., Sevdalis N., Vincent C.A. (2012). Patient involvement in patient safety: the health-care professional's perspective. J. Patient Saf..

[28] Burrows Walters C., Duthie E. (2017). Patient engagement as a patient safety strategy: patients' perspectives HHS public access. Oncol. Nurs. Forum.

[29] Liang L., Cako A., Urquhart R., Straus S.E., Wodchis W.P., Ross Baker G., Gagliardi A.R. (2018). Patient engagement in hospital health service planning and improvement: a scoping review. BMJ Open.

[30] Sharma A.E., Rivadeneira N.A., Barr-Walker J., Stern R.J., Johnson A.K., Sarkar U. (2018). Patient engagement in health care safety: an overview of mixed-quality evidence. Health Aff..

