# Supplementary material for: How to engage patients in achieving patient safety: A qualitative study from healthcare professionals’ perspective
Source: Heliyon. 2023 Feb 7;9(2):e13447. doi: 10.1016/j.heliyon.2023.e13447 (PMC9946844; doi:10.1016/j.heliyon.2023.e13447)
Supplement: Multimedia component 1 [file mmc1.docx]

COREQ (COnsolidated criteria for REporting Qualitative research) Checklist

| **Topic** | **Item No.** | **Guide Questions/Description** | **Reported on**  **Page No.** |
| --- | --- | --- | --- |
| **Domain 1: Research team**  **and reﬂexivity** | | | |
| *Personal characteristics* | | | |
| Interviewer/facilitator | 1 | Which author/s conducted the interview or focus group? | Method – data analysis – page 5 |
| Credentials | 2 | What were the researcher’s credentials? E.g. PhD, MD | Method – data analysis – page 5 |
| Occupation | 3 | What was their occupation at the time of the study? | Method – data analysis – page 5 |
| Gender | 4 | Was the researcher male or female? | Method – data analysis – page 5 |
| Experience and training | 5 | What experience or training did the researcher have? | Method – data analysis – page 5 |
| *Relationship with participants* | | | |
| Relationship established | 6 | Was a relationship established prior to study commencement? | Method – ethics – page 5 |
| Participant knowledge of  the interviewer | 7 | What did the participants know about the researcher? e.g. personal goals, reasons for doing the research | Method – ethics – page 5 |
| Interviewer characteristics | 8 | What characteristics were reported about the inter viewer/facilitator?  e.g. Bias, assumptions, reasons and interests in the research topic | Method – ethics – page 5 |
| **Domain 2: Study design** | | | |
| *Theoretical framework* | | | |
| Methodological orientation and Theory | 9 | What methodological orientation was stated to underpin the study? e.g. grounded theory, discourse analysis, ethnography, phenomenology,  content analysis | Method – study designs – page 3 |
| *Participant selection* | | | |
| Sampling | 10 | How were participants selected? e.g. purposive, convenience,  consecutive, snowball | Method – participants – page 3 |
| Method of approach | 11 | How were participants approached? e.g. face-to-face, telephone, mail,  email | Method – participants – page 3 |
| Sample size | 12 | How many participants were in the study? | Method – participants – page 3 |
| Non-participation | 13 | How many people refused to participate or dropped out? Reasons? | Method – participants – page 3 |
| *Setting* | | | |
| Setting of data collection | 14 | Where was the data collected? e.g. home, clinic, workplace | Method – data collection – page 4 |
| Presence of non-  participants | 15 | Was anyone else present besides the participants and researchers? | Method – data collection – page 4 |
| Description of sample | 16 | What are the important characteristics of the sample? e.g. demographic  data, date | Method – participants – page 3 – 4 |
| *Data collection* | | | |
| Interview guide | 17 | Were questions, prompts, guides provided by the authors? Was it pilot  tested? | Method – data collection – page 4 |
| Repeat interviews | 18 | Were repeat inter views carried out? If yes, how many? | Method – data collection – page 4 |
| Audio/visual recording | 19 | Did the research use audio or visual recording to collect the data? | Method – data collection – page 4 |
| Field notes | 20 | Were field notes made during and/or after the interview or focus group? | Method – data collection – page 4 |
| Duration | 21 | What was the duration of the inter views or focus group? | Method – data collection – page 4 |
| Data saturation | 22 | Was data saturation discussed? | Method – data analysis – page 5 |
| Transcripts returned | 23 | Were transcripts returned to participants for comment and/or correction? | N/A.  Professional transcribers transcribed the interviews and researchers checked for accuracy |
| **Domain 3: analysis and**  **ﬁndings** | | | |
| *Data analysis* | | | |
| Number of data coders | 24 | How many data coders coded the data? | Method – data analysis – page 5 |
| Description of the coding  tree | 25 | Did authors provide a description of the coding tree? | Results – page 10 |
| Derivation of themes | 26 | Were themes identified in advance or derived from the data? | Method – data analysis – page 5 |
| Software | 27 | What software, if applicable, was used to manage the data? | Method – data analysis – page 5 |
| Participant checking | 28 | Did participants provide feedback on the findings? | N/A  However, participants were received a report of this study and able to give a feedback on the findings. |
| *Reporting* | | | |
| Quotations presented | 29 | Were participant quotations presented to illustrate the themes/findings?  Was each quotation identified? e.g. participant number | Results – page 7 – 9 |
| Data and findings consistent | 30 | Was there consistency between the data presented and the findings? | Results – page 6 – 9 |
| Clarity of major themes | 31 | Were major themes clearly presented in the findings? | Results – page 6 |
| Clarity of minor themes | 32 | Is there a description of diverse cases or discussion of minor themes? | Results – page 6 – 9 |

Developed from: Tong A, Sainsbury P, Craig J. Consolidated criteria for reporting qualitative research (COREQ): a 32-item checklist for interviews and focus groups. *International Journal for Quality in Health Care*. 2007. Volume 19, Number 6: pp. 349 – 357
